# Demography and Causes of Mortality of Pugs Under Primary Veterinary Care in Australia

**DOI:** 10.3390/vetsci12030195

**Published:** 2025-02-21

**Authors:** Karmen Wong, Peter Williamson, Rosanne M. Taylor

**Affiliations:** Sydney School of Veterinary Science, The University of Sydney, Sydney, NSW 2006, Australiap.williamson@sydney.edu.au (P.W.)

**Keywords:** epidemiology, electronic patient records, euthanasia, longevity, VetCompass

## Abstract

Pugs are a popular brachycephalic breed that suffer from multiple chronic disorders linked to their exaggerated phenotypic traits. The contribution of these disorders to early death and euthanasia have not been described and would add urgency to addressing these issues. This study used electronic patient records (EPR) from the Australian VetCompass programme to describe the demography, common causes and risk factors for Pug mortality. The EPR from 691 Pugs which died in a population of 7909 Pugs that received veterinary care over a 10-year period were analysed to determine the cause of death. The median age at death was 10 years. Male Pugs had lower probability of surviving than females (*p* = 0.02) and entire Pugs died earlier than neutered dogs. The top causes of mortality were Brachycephalic Obstructive Airway Syndrome (8.2%), seizures (6.7%) and degenerative spinal cord disorder (4.7%). Neurological causes (29.6%) were the most common cause of euthanasia whilst amongst non-assisted deaths, respiratory causes were most common (25%). Death from respiratory disorders was found across all age groups. These neurological and respiratory causes of death are linked to brachycephalic conformation and provide evidence for reform of showing and breeding standards to improve Pug welfare and longevity.

## Simple Summary

Pugs are a popular brachycephalic (flat-faced, short-muzzled) breed that suffer from multiple chronic disorders linked to their exaggerated shape. These disorders are common and cause suffering. However, their contribution to early death and euthanasia have not been thoroughly studied. This study used electronic patient records from the Australian VetCompass programme to describe the demography, common causes and risk factors for Pug mortality. The records of a large group (691) of Pugs who died while under veterinary care over a 10-year period were analysed to determine the cause of death. The median age at death was 10 years. Male Pugs had shorter median lifespans than females, and entire Pugs died earlier than neutered dogs. The top causes of mortality were Brachycephalic Obstructive Airway Syndrome, seizures and degenerative spinal cord conditions, which are uncommon in breeds with a normal head shape. The most common reason for euthanasia was neurological causes, whilst for spontaneous deaths, respiratory causes were the most common. These neurological and respiratory causes of death are linked to brachycephalic conformation and provide evidence for reform of showing and breeding standards to improve Pug welfare and longevity.

## 1. Introduction

The origin of the Pug dates back to Ancient China, where selective breeding for the flat-faced appearance was favoured by the royal family. Pug, from the Greek word “Pugnus”, means fist and alludes to the flat, fist-like appearance of the Pug’s side profile [1]. The breed was first recognised by the American Kennel Club (AKC) in 1885, and its popularity has grown considerably in recent years [2,3]. In 2017, they were the 11th most popular registered breed in Australia from the annual national registration data of the Australian National Kennel Club.

Despite the well-documented health issues of the Pug, demand for the breed continues to rise. Characteristics of the Pug, such as large, round eyes, short muzzle and attention-seeking behaviour, resemble anthropomorphic infant traits that are endearing to humans [4]. This phenomenon, known as neoteny, elicits a nurturing response from humans and is seen as more attractive, especially to a female audience [4]. However, substantial evidence has linked exaggerated brachycephalic traits seen in Pugs to chronic health disorders [3]. The contribution of brachycephalic-related conditions to cause of death has not yet been reported in Pugs. In response to rising concerns regarding conformation-related health and welfare problems in the breed, the Australian breed standard for Pugs has been revised to reduce the emphasis on exaggerated features such as large, prominent eyes, which were described in the previous breed standard [5]. Pug breeders are encouraged to use genetic and imaging tests for recognised problems in the breed. While this demonstrates some effort in mitigating health disorders in Pugs, further action is needed as disorders are still prevalent in the breed, prompting the Australian Veterinary Association’s policy calling for stronger action on breeding dogs with brachycephalic disorders [6]. A 2016 UK VetCompass study analysing primary data collected from 2013 veterinary patient records in the UK revealed that 68.19% of Pugs had at least one disorder recorded, the most prevalent being obesity (13.18%), corneal disorder (8.72%) and otitis externa (7.53%) [3]. A major disorder that was ranked 8th most common in the UK VetCompass Pug study was Brachycephalic Obstructive Airway Syndrome (5.15% prevalence) (BOAS). Pugs had 53.9 times greater risk for this condition, which impacts health and welfare compared to non-Pugs [7,8,9]. Using a functional grading system to determine the severity of BOAS, Ladlow et al. (2018) found that 50% of Pugs had clinically significant symptoms [8]. BOAS is a serious disorder that greatly impedes the Pug’s ability to participate in normal exercise activities, interferes with sleep, exacerbates the risk of heat stroke and predisposes Pugs to obesity [8,9]. Obesity contributes to a myriad of secondary diseases, such as osteoarthritis, cardiopulmonary disease, glucose intolerance, dysfunction of multiple organ systems, and diabetes mellitus [10]. Therefore, while BOAS may not be immediately life-threatening, it has major health implications for the quality of life of Pugs, reducing their ability to exercise, compromising sleep quality and can become progressively worse in later years. Despite this, BOAS is often insufficiently recognised and has become normalised as a feature of brachycephalic breeds by owners and veterinarians [11,12]. A survey study in 2019 found that despite owners’ awareness of their dog’s health issues, 70.9% considered their dog to be in very good health [13,14]. The recent efforts to reduce the impact of brachycephalic conformation have not had sufficient impact, so greater action in addressing the health issues affecting Pugs is needed. Seemingly harmless disorders, which may be considered endearing in puppies, such as snuffling and snoring, can have a significant impact on the quality of life in Pugs and contribute to early euthanasia decisions.

In contrast to the many studies of the disorders affecting Pugs, the causes of mortality are poorly documented. A VetCompass study found that neoplasia (16.5%), musculoskeletal (11.3%) and neurological (11.2%) disorders were the most common, broad causes of mortality in the general dog population within the UK. These results were supported by mortality studies undertaken in other countries [14,15,16,17,18], however, they did not report causes of mortality or describe the contribution of brachycephalic abnormalities to premature death in Pugs. Understanding breed-related causes of mortality is important as individual dog breeds are associated with certain disorders and thus, it can be expected that there are also breed-related causes of mortality. Flemings et al.’s (2011) study in North America demonstrated this at the body system level; the study’s investigation of Dutch Pugs found that neurological diseases (organ system) and neoplastic causes (pathophysiological processes) contributed to the largest proportion of deaths in the breed [18]. This study only used broad categories for the causes of death and specific disorders were not determined. Therefore, a breed-specific study of the causes of mortality in Pugs is necessary to identify the contribution of brachycephalic disorders to early death and euthanasia.

Pugs have a breed predisposition to neurological disorders, particularly seizures and spinal cord disorder conditions, which are likely to contribute to mortality. A UK VetCompass study found that Pugs had the highest prevalence of seizure disorders within the canine population [19]. Among Pugs attending a specialist neurology clinic in the UK, the major diagnoses were myelopathy (64.7%), including intervertebral disk disease, spinal arachnoid diverticulum and vertebral malformations; and encephalopathy (29.7%), including idiopathic epilepsy and necrotising meningoencephalitis (NME) [20]. NME is a progressive inflammatory central nervous system disorder that can cause seizures and a range of other signs related to myelopathy and encephalitis. Young Pugs are at increased risk of this progressive, fatal condition, with multifocal neurological signs including seizures, blindness, gait deficits and ataxia. The AKC reported that 1.2% of Pugs die from NME [1]. A variant CFA12:2605517delC has been identified to increase risk in Pugs [21], along with other associations [22]. Idiopathic epilepsy is a common encephalopathy in the breed [20]. Pugs are predisposed to cluster seizures, which can be distressing for both the dog and the owner and have been associated with increased likelihood of euthanasia compared to single episodes.

Most Pugs (96%) have congenital vertebral malformations, including ventral wedge shape vertebrae, shortened vertebrae, hemivertebrae, caudal articular process dysplasia, lumbosacral transitional vertebrae, kyphosis, spina bifida and other abnormalities such as intervertebral disc disease [23,24,25], which may cause clinical signs of myelopathy. Mutations contributing to skeletal malformations have been identified in other brachycephalic breeds (*SMOC2*, *BMP3*); however, Pugs did not have the *DVL2* mutation associated with brachycephaly in other breeds [21,26]. While these vertebral abnormalities can be detected by radiological examination and scored for severity in dogs that do not have neurological signs at that time, Pugs more frequently develop clinical signs such as urinary incontinence, ataxia of pelvic limbs, pain and paraparesis than other screw-tailed brachycephalic breeds with these abnormalities [23,25,27]. Although these studies did not report on mortality, the high frequency of vertebral abnormalities, neurological signs, painful episodes, and the progressive degenerative aspects of these disorders are likely to impact the quality of life and contribute to euthanasia or death.

Pugs have been reported to have a predisposition to mast cell tumours (MCT), although typically benign and of low to intermediate grade [28,29]. An American Veterinary Medical Database study reported 3 deaths (12%) out of 25 purebred Pugs with MCT [28], however, the contribution of MCT to mortality in the breed is not known.

The aim of this study was to identify the most common causes of mortality in Pugs using a retrospective cohort study utilising electronic patient records (EPR) from the Australian VetCompass programme [30]. All records of death in Pugs recorded in 10 years of VetCompass Australia data were examined to determine the method and causes of death. Demographic risk factors for the most common causes of mortality were determined. The findings identify the significant health issues contributing to mortality in Pugs. This can inform breed-specific health management strategies and provide evidence for further changes to the breed standards.

## 2. Materials and Methods

### 2.1. Ethics Statement

The study was approved by the University of Sydney Human Research Ethics Committee—VetCompass Australia (project number: 2013/919)

### 2.2. Data Source

This research was undertaken with the assistance of information and other resources from the VetCompass Australia consortium under the project “VetCompass Australia: Big Data and Real-time Surveillance for Veterinary Science”, supported by the Australian Government through the Australian Research Council LIEF scheme (LE160100026) [30]. VetCompass is a programme that collects real-time de-identified patient records directly from participating veterinary clinics across Australia and enters it into a centralised database. Information collated includes general patient demographic (species, breed, colour, sex, date of birth, deceased date, neuter status and body weight) and clinical information (free-form examination texts and any relevant treatment administered or prescribed).

### 2.3. Statistical Analysis

#### 2.3.1. Demography

The study undertakes a retrospective, cohort approach, investigating the demography, longevity and causes of mortality in Pugs in Australia [31]. Demographic risk factors associated with disease was also explored. The sampling frame included all dogs, which were classified as a Pug under veterinary care at participating clinics within the VetCompass Programme [30] between 1 January 2008 and 31 December 2017. Pugs under veterinary care were identified by having at least one electronic patient record (EPR) within the study period.

Demographic analysis was completed for all Pugs presented to participating VetCompass practices in Australia (*n* = 7911) and for a subset of Pugs that had a date of deceased record (*n* = 691) within the 10-year period. Data were checked for validity and cleaned in Excel (Microsoft Office Excel 2009., Microsoft Corp.). Dogs were excluded if the recorded date of birth was after the consultation date. Within each demographic variable, inaccurate records were further excluded (e.g., weight recorded as a monetary value; sex not recorded as either female or male). Due to the wide range of colours recorded for Pugs in the dataset, colour was reclassified into 6 colours (fawn, black, silver, apricot, brown and brindle) for easier comparison. Age was grouped in 5 groups (<3, 3–5.9, 6–8.9, 9–11.9, ≥12) and bodyweight data were also grouped into 5 groups (0–3.9 kg, 4–7.9 kg, 8–11.9 kg, ≥12, not available {N/A} and 1 kg intervals). The annual national registration data from the ANKC was also collated. Frequency distribution and proportions were calculated for all demographic variables in Excel.

#### 2.3.2. Bodyweight Model

Pugs were selected for inclusion in a study of normal bodyweight in the breed. Dogs were filtered for wellness checks and were excluded if they were recorded as being underweight, overweight or obese. The following words and abbreviations were used to filter for wellness checks: *vacc, chip, spey, desex, vaccination, vaccine, vax, protech, c3, c4, c5, nail clip, proheart, spay, spey, castrate, desexing, tattoo, wellness, gland express, puppy cadets, c7, duramune, castration, heartworm injection*. All recorded weights for Pugs equal to or under the age of 12 months were included, with exemption of abnormal weights (e.g., 1-month Pug weighing 30 kg). The ANKC breed standard for Pugs is 6.3 kg–8.1 kg, therefore, the feasible weight range for adult Pugs was determined by half the minimum and double the maximum breed standard weight (3.15 kg–16.2 kg). Any bodyweight data outside of the feasible weight range were excluded for Pugs above the age of 12 months. Pugs without a weight record or an incorrect weight record were further excluded from the normal bodyweight study. Bodyweight curves followed the methods of Tambalis et al. [32]. In RStudio, the LMS function was used to model a growth curve for female and male Pugs. Centile estimations at the 5th, 25th, 50th, 75th and 95th percentiles were displayed.

#### 2.3.3. Longevity and Mortality Study

All Pugs with recorded deceased data were included in the longevity study. The clinical notes of individual dogs were analysed by the researchers for the cause of death. A corresponding VeNom code [33] was assigned at the most specific fine-level diagnosis. VeNom codes are a list of standardised codes, which correspond to specific diagnoses, presenting complaints or procedures. Dogs were also given a general cause of death: behavioural, traumatic, disorder, advanced age or unspecified. Top-level body system groupings were also used to classify disorders in Pugs (e.g., seizure disorder is classified into the neurological top-level grouping). Mode of death was grouped as either dogs that died without euthanasia or dogs that were euthanised or unspecified.

#### 2.3.4. Statistical Analysis

Data were analysed using RStudio (v 1.2.1335) and Excel (Microsoft Excel 2009). The frequency and prevalence of fine-level diagnosis of disorders, group-level disorders and causes of death were calculated in Excel. Univariate and multivariate generalised linear models were used to analyse the correlation between demographic factors (sex, age, weight, neuter status and colour) and the presence of major disorders at the group level. The risks associated with demographic factors were first analysed using a univariate model. Body systems with more than two significant explanatory variables were further tested using a multivariate model to determine if there was an interaction effect. *p*-values of less than 0.05 and 95% confidence interval were used to determine statistical significance. Kaplan–Meier survival function was generated for survival analysis between male and female Pugs.

## 3. Results

### 3.1. Demography of Population

**Figure 1:**
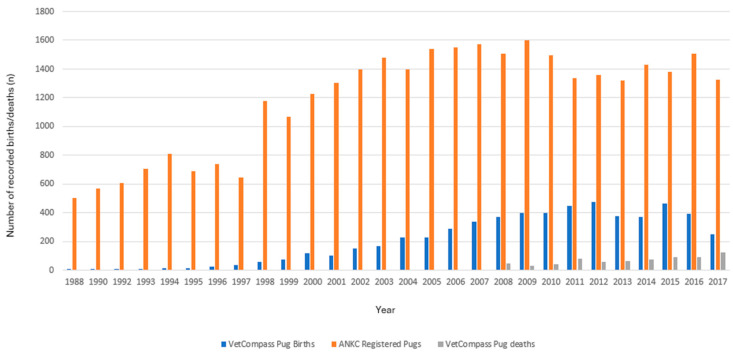
Number of births and deaths recorded from participating VetCompass clinics in Australia between the 1 January 2008 to 31 December 2017 plotted against the ANKC national registrations of Pugs for the corresponding years.

The study was comprised of 115,117 EPR records for 7909 Pugs within the study period. The records of all 691 Pugs (8.7%) who died during the 10-year study period were analysed. Annual birth rates showed a steady increase in the annual VetCompass birth cohort between 2002 and 2012 (based on date of birth data from the sampled population), followed by a slight decline (Figure 1).

A description of the demography of the whole Pug population and the subset of deceased Pugs within the 10-year study period is summarised in Table 1. The whole Pug population had more female (*n* = 4085, 51.65%) than male Pugs (*n* = 3780, 47.79%) whilst in the deceased population, there were slightly more male (*n* = 350, 50.65%) than female Pugs (*n* = 340, 49.29%). There were more neutered Pugs than entire Pugs for both sexes (Table 1). In the whole Pug population, the largest group of Pugs attending veterinary care were <3 years of age, (*n* = 3653, 46.19%) whilst in the deceased population, the largest group was Pugs over 12 years old (*n* = 243, 35.17%). Most Pugs were in the 8–11.9 kg weight group where weight was recorded in the EPR (whole population: *n* = 2354, 29.8%; deceased population: *n* = 234, 33.9%). The most common coat colour recorded in the Pug population was fawn (60.45%), followed by black (21.52%), brown (11.42%), apricot (1.73%), silver (0.44%) and brindle (0.33%).

**Figure 2:**
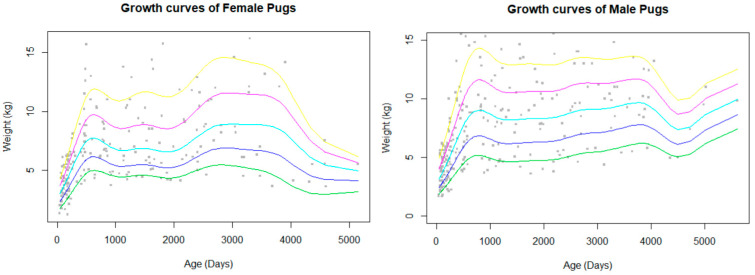
Bodyweight curves with percentile estimations (5% green, 25% blue, 50% aqua, 75% pink, 95% yellow) for female and male Pugs. Body weight data only included Pugs that visited primary-care veterinary practices for wellness visits and routine checks.

The bodyweight data available for analysis were limited to a small group of Pugs which met the criteria for inclusion (attending wellbeing visits, not overweight or obese), so only a total of 471 bodyweight values were included out of a total of 115,117 potential EPRs. This comprised of 393 dogs (260 male records and 211 female records) giving a data completeness of 0.05%. The bodyweight curves showed a rapid growth during the first year of life, which slowed before it plateaued and was maintained until decline in old age (Figure 2). Males had a higher peak bodyweight than females. The median bodyweight across all ages, including puppies, for males (6 kg, IQR: 4.4–9.5, range: 1.6–16.2) was similar to females (6.09 kg, IQR: 4.3–8.2, range: 1.2–16.2).

### 3.2. Mortality Analysis

**Figure 3:**
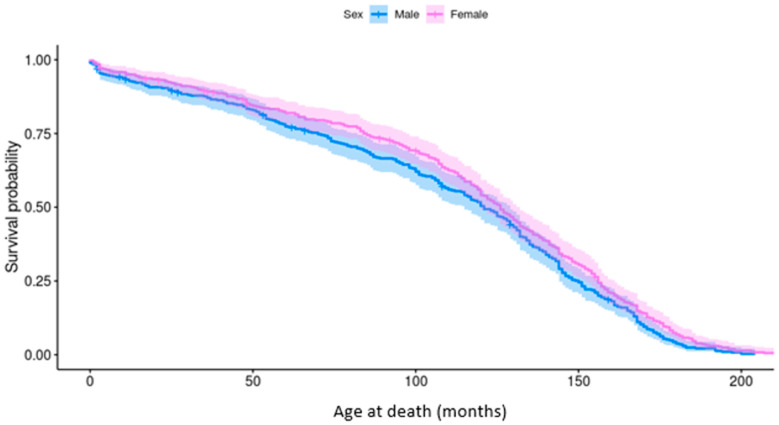
Kaplan–Meier survivor function generated for survival analysis of male and female Pugs attending wellness visits. The cumulative survival probability is shown against time of death (months) with confidence intervals.

The longevity of Pugs was bimodally distributed; peaks were present at <1 year and between 10 and 12 years of age. The median longevity was 10 years old for both females (IQR: 6–12; range: 0–17) and males (IQR: 7–13; range 0–18). Neutered Pugs (11 years; IQR: 8–13; range: 0–18) lived longer than entire Pugs (8 years; IQR: 2–6; range: 0–17), however age at neutering was not reported. Males had a significantly lower probability of survival than females (*p* = 0.03), particularly between the ages of 4 and 10 years (Figure 3).

Among the 491 Pugs that had a diagnosis for cause of death, 58.3% of Pugs were euthanised, 20.4% died non-assisted, and for the remaining Pugs, the mode was not reported. The most prevalent causes of all mortality at the finest (most specific) level of diagnostic precision were BOAS (*n* = 40; 8.2%), seizure disorder (*n* = 33; 6.7%), unspecified neurological disorder (*n* = 22; 4.5%), degenerative spinal cord disorder (*n* = 23; 4.7%), and cognitive dysfunction (*n* = 21; 4.3%). Among euthanised Pugs, BOAS (*n* = 29; 8.1%), seizure disorder (*n* = 25; 7.0%) and degenerative spinal cord disorder (*n* = 20; 5.6%) were the top reasons for euthanasia. The top causes of non-assisted deaths were BOAS (*n* = 8; 7.2%), seizure disorder (*n* = 8; 7.2%), heat stroke (*n* = 7; 6.3%), respiratory failure (*n* = 5, 4.5%) and gastroenteritis (*n* = 4, 3.6%) (Table 2).

The most prevalent group-level precision (broader classification) disorders for euthanised Pugs were neurological (*n* = 106; 29.6%), mass lesion (*n* = 57; 15.9%) and respiratory (*n* = 53; 14.8%). For Pugs that died non-assisted, respiratory (*n* = 28; 25.0%), traumatic injury (*n* = 15; 13.4%) and neurological (*n* = 14; 12.5%) were the main causes of death at the group-level (Table 3). For Pugs that died when they were less than 1 year old, traumatic injury was the top cause of mortality. Neurological, mass lesion and respiratory disorders were prevalent across all ages.

### 3.3. Signalment Factors

Neurological disorders at death were associated with age (*p* = 0.049; CI = 1, 1.013). Respiratory causes were not associated with any demographic variables. In the univariate analysis, deaths associated with mass lesions were associated with neutering (*p* <= 0.01; CI: 1.03–1.14), age (*p* < 0.01; CI: 1.00–1.01) and weight increments (*p* < 0.01; CI: 1.00–1.02) however in the multivariate analysis, neuter status was no longer significant and an interaction effect was seen between age and weight increments (*p* = 0.02). Death caused by alimentary tract disorders was associated with age (*p* = 0.03; CI: 0.99–1.0) and weight increments (*p* = 0.03; CI: 0.99–1.0) in the univariate analysis (Table A1), however in the multivariate analysis, only weight was significant (*p* = 0.04) (Table 4). Male Pugs had an odds ratio of 1.03 to female Pugs of dying from musculoskeletal disorders (*p* = 0.044; CI = 1.001–1.061) (Table A1). An interaction effect was seen between neuter status and age (*p* = 0.007) for Pugs that died from traumatic injury (Table 4). Pugs that were not neutered died at a younger age from traumatic injury than Pugs that were neutered.

## 4. Discussion

This study provides the largest and most comprehensive analysis of the causes of mortality in Pugs based on veterinary practice records to date. BOAS was the leading cause of mortality in Pugs for both euthanasia and unexpected deaths. This is an important finding as BOAS has often not been considered to be immediately life-threatening [8] and had not been documented as the major cause of Pug mortality, despite its prevalence worldwide. The BOAS VeNom code was assigned to cases with clinical signs of respiratory difficulty, dyspnoea, laryngeal collapse and upper respiratory tract obstruction before death. Despite the progressive nature of BOAS [8], it was unexpected to find that there was no association between age and respiratory causes of death, revealing that death due to respiratory complications is a risk for Pugs across all life stages. This contrasted with neurological disorders (including seizures and spinal cord disease), and mass lesions, which were reported in older age groups and traumatic injury, which was associated with younger age groups.

Most of the respiratory causes of Pug mortality were directly related to BOAS. These may be underrepresented in the study, because dogs that died unexpectedly at home, e.g., from respiratory collapse or heat stress, were less likely to be presented to veterinary practices. Brachycephalic breeds with respiratory obstruction, sleep apnoea, poor oxygenation and regurgitation are at greater risk during anaesthetic induction, extubation and recovery [31,34]. This is consistent with the current study’s findings as anaesthetic death due to respiratory complications was a frequent cause of death in the study (4.5%). Due to a lack of a VeNom code, these cases were reclassified as respiratory failure, which was the 4th major cause of non-assisted death in Pugs (Table 2). Another major cause of death that was exacerbated by brachycephalism was heat stroke, which was responsible for 11 deaths in the study and ranked 6th in the overall causes of mortality. Due to nasal and pharyngeal limitations in capacity for dissipating heat through panting [8], death by heat stroke was classified at the top-level grouping as a respiratory cause. A retrospective study by Bruchim et al. (2006) of 54 cases of heat stroke in dogs found that obesity and seizures were significant risk factors for death [35], and these two disorders are prevalent in the Pug breed [3,19]. An adverse relationship is seen between BOAS and obesity as a reduced ability of Pugs to exercise due to BOAS increases weight gain, and the accumulation of adipose deposits further exacerbates the effects of BOAS. Other unexpected causes of death such as drowning (2.7%) are rarely reported in dogs and may be related to the frailty of respiratory function in this breed.

The impact of BOAS on respiratory function can be quantitatively assessed using whole-body barometric plethysmography [36,37]. A functional grade of 0 or 1 represents mild cases of BOAS with little impact on the quality of life, while more severe gradings require surgical intervention [36,38]. While surgical treatment alleviates the severity of airway restriction, the mortality rate after treatment is 4% [39], and normal function is not restored. Future investigation of the long-term impacts of mild BOAS in Pugs will determine whether the prolonged impact of hypoxia and hypertension contribute to a decline in quality of life as Pugs age. Veterinarians advise that only Pugs that are free of clinical symptoms of BOAS should be used in breeding [6] and should ensure owners maintain a normal BCS in Pugs with BOAS to reduce the health impacts of this condition.

At the group-level diagnostic precision, neurological and mass lesion disorders were the most common causes of mortality [8,40]. This was consistent with Flemings et al.’s (2011) study which found that the leading organ system responsible for Pug mortality was the nervous system while neoplasia was the most prevalent pathophysiological cause of death [18]. Their study did not define the causes of Pug death at a fine level of diagnosis, and this gap has been addressed by the current study.

Seizure disorder and degenerative spinal cord disorder were the most prominent neurological causes of mortality in this study. A VetCompass study in the UK investigating seizure occurrence in dogs found that Pugs had the highest prevalence of seizures amongst all dog breeds [19]. In the current study, clinical notes that described Pugs as having epilepsy, seizures, fits or convulsions without other diagnoses were classified as having seizure disorder. Many Pugs in the study had reoccurring episodes of seizures, which informed decisions to euthanise. Morphological deformities associated with brachycephaly and skull foreshortening may lead to the accumulation of cerebrospinal fluid and cerebral compression due to flow obstruction. These changes in the skull increase the risk of seizures and neurological decline with aging due to impairments in brain development, nutrition, and function [31]. Pugs have a breed predisposition for NME, with up to 94% of Pugs that are homozygous for the mutant alleles experiencing seizures, however there is an additional mutation on chromosome 8 [21]. Testing is available for the NME chromosome 12 mutation, and European Pugs had a 7.4% frequency of homozygosity, with many, but not all, homozygous Pugs having neurological signs at testing. However, most EPR in the current study did not differentiate the cause of seizures in Pugs or report on testing for NME, suggesting it is under-recognised by breeders and veterinarians in Australia. The “screw-tail” phenotype is highly associated with sacrocaudal malformations, kyphosis and scoliosis, which contribute to myelopathy in other brachycephalic breeds. Furthermore, Pugs have been shown to have an increased risk of developing clinical signs compared to other screw-tailed brachycephalic breeds [23,27]. The euthanasia deaths due to intervertebral disc disease (2%) and some urinary conditions (incontinence) may have also been related to myelopathy. The breed risk for neurological and BOAS-related conditions is substantial, and efforts to identify dogs free of disease for breeding may severely restrict the gene pool, particularly due to the relative isolation of the Pug population in Australia. Therefore, Pug health may benefit from strategic outcrossing to other breeds and selecting unrelated sires from other countries [19]. Breeding for longer skulls and spines and reducing or removing the breed standard emphasis on the screw-tail phenotype, which is associated with vertebral anomalies, should help to reduce the prevalence of neurological disorders over time [31].

Within the neoplastic causes of mortality, mast cell tumour and lymphoma were major causes of death or euthanasia. This is consistent with previous studies that reported a breed predisposition to mast cell tumours (MCT) however, MCT is often low grade in Pugs. On the contrary, an Australian epidemiological study found that Pugs had a decreased risk of lymphoma [41]. Most Pugs in the study with neoplasia or mass lesions were euthanised but the severity of the malignancies and precise pathological diagnoses were often not stated. For example, the findings of needle aspirate examinations for tumour cell morphology were infrequently reported which suggested that limited or no cytological or histopathological diagnosis was undertaken. This limited the analysis and thus, most disorders in this group were either classified as neoplasm or mass lesion. Primary care veterinarians can improve diagnosis, prognosis and management of MCT and lymphoma by consistent use of fine needle aspirate and biopsy for pathological diagnosis [42].

The median longevity in this study is 10 years, which is shorter than the median longevity of Pugs in other studies. A survey study found the median longevity of Pugs to be 11 years [14]. While survey studies are subject to recall biases, in a Japanese study which used cemetery data, the median longevity was 12 years [43]. The current study spanned a decade in which the Pug population grew, mostly due to new puppies (Figure 1) [4]. Pugs under the age of 1 year died mostly from traumatic injury. Given the young population structure of this study, a considerable number of Pugs that were still alive and expected to live longer than the deceased population were not included in the longevity analysis, as well as unreported home deaths and thus, the data are right censored. A median longevity of 11 and 12 years has been previously reported for dogs overall in several studies [14,16,44,45]. Pugs also have a higher median longevity than most brachycephalic breeds; bulldogs have a median longevity of 7 years, and for French bulldogs, it is about 9 years [14]. While the lifespan of Pugs is not far from the average lifespan of dogs, efforts to mitigate disease in Pugs are vital to improve their quality of life.

Pugs were most often euthanised for health disorders, with only 3.2% of Pugs being euthanised for old age and 5.6% for cognitive decline. Among the deceased dogs, 58.3% of Pugs were euthanised, and 82.4% of these were due to disorders or health conditions. A higher percentage of euthanasia (86.4%) was reported in a mortality study of dogs in England [16]. The EPRs of 21.3% of Pugs in the current study did not specify a mode of death, and therefore, the percentage of Pugs that were euthanised in the current study may be underestimated. Studies of the reasons for euthanasia similarly reported illness to be the most common reason, followed by senility [46,47].

Limited description and the reliance on researcher interpretation of brief notes were limitations experienced in this study, and other studies using EPR data. Often clinical notes lacked clinical examination findings detail (e.g., body condition score, specific neurological deficits) or reports of simple clinical investigations (e.g., fine needle aspirates) so specific diagnoses could not be determined. A large proportion of neurological disorders were unspecified, and further diagnostic imaging was usually not performed; thus, diagnoses was based on neurological examination findings and clinical history. Euthanasia consultations and death records from young dogs often recorded few to no clinical details, and while the researchers considered the recent, relevant clinical history, lack of detail at the time of death may have resulted in some disorders being underestimated. In the case where no diagnosis was recorded, the most likely diagnosis was determined by analysing all EPR notes and the treatment offered or provided for individual Pugs. Interpretation of clinical notes may differ between researchers, so difficult cases were cross-checked with a second experienced veterinarian. This system may be improved by including VeNom codes in the software used in veterinary practices as prompts for diagnoses. This should encourage veterinarians to provide a specific diagnosis. However, this was completed in the UK VetCompass programme, and compliance rates were still fairly low [3]. It is notable that the major causes of death identified by this study are consistent with the reports of the most common, severe health conditions reported in Pugs using specialist centre data overseas and pet insurance data from Australia [31].

This study used stringent parameters for the development of the growth curves, with data selection limited to the body weights available from healthy Pugs. Only Pugs that were under veterinary care for wellness visits were included by filtering for specific abbreviations or words in Excel. Pugs with disorders that may influence their body weight were excluded. This resulted in an analysis of only 470 EPRs from the total 115, 117 EPRs (0.4%) over the 10-year period and a median body weight (all ages) of 6 kg. Other canine growth curves were calculated with a broad population sample and did not exclude dogs who may have been overweight or unwell [48]. The limited data points in our study demonstrate how few Pugs are disease-free across all stages of their lives. BOAS problems caused mortality in Pugs across all life stages, and most other disorder groups were significantly associated with age. Obesity was the most prevalent condition (13.18%) in the UK Vet Compass study [3], and an assessment of show dogs found that more than 80% of Pugs were considered to be overweight [12]. The median bodyweights of the deceased dogs in the current study were 8–11.9 kg, higher than the ANKC breed standard weight of 6.3–8.1 kg [5]. However, the current study does not represent solely purebred Pugs. Breed classification in the VetCompass database is reliant on the input of veterinarians, and thus, dogs which are phenotypically similar to Pugs, or identified by their owners to be Pugs, were included. This was also seen in the UK VetCompass study, where body weights were considerably above breed standard weights. Finally, the age at neutering was not consistently recorded. Therefore, the effects of the duration of neuter status on mortality were not evaluated.

The recent increase in Pug ownership has not been accompanied by better community understanding of the health problems in this breed. Veterinarians should implement health care and client education specific for Pugs that is targeted at screening for BOAS, NME and vertebral abnormalities, early diagnosis and energetic management. Owners should be made more aware of the health and welfare risks of extreme brachycephaly by the veterinary profession working in conjunction with breeders and their kennel club [31].

## 5. Conclusions

This study supports the need to prioritise early diagnosis and active management of the common conditions which contribute to death in Pugs. The analysis of 7909 Pugs attending primary care veterinary clinics over a 10-year period provided demographic information and described the major causes of death in 691 Pugs who died. The most common causes of mortality in Pugs at the diagnostic level were BOAS, seizures and spinal cord disorder. At the group level, neurological, respiratory, and musculoskeletal disorders were the most prominent causes of mortality. Death from respiratory disorders was prominent across all age groups, and the main cause of death under the age was traumatic injury. These results highlight priorities in Pug health and can inform future reforms to improve welfare and increase longevity in the breed.

## Figures and Tables

**Table 1:** Demography of the Pug population and the subset population of deceased Pugs under primary veterinary care at participating VetCompass clinics in Australia between 1 January 2008 and 31 December 2017.

		All Pugs		Pugs that Died	
Variable	Category	Count	Percent (%)	Count	Percent (%)
Sex	Male	3780	47.8%	350	50.7%
	Female	4085	51.7%	340	49.3%
	Unspecified	44	0.6%	1	0.1%
Neuter status (Male)	Entire	1611	20.4%	107	15.5%
	Neutered	2474	31.3%	233	33.7%
Neuter status (Female)	Entire	1488	18.8%	94	13.6%
	Neutered	2292	29.0%	256	37.0%
Colour	Fawn	4781	60.5%	441	63.8%
	Black	1701	21.5%	119	17.2%
	Apricot	137	1.7%	14	2.0%
	Silver	35	0.4%	1	0.1%
	Brown	903	11.4%	76	11.0%
	Brindle	26	0.3%	0	-
	NA	326	4.1%	41	5.8%
Age	<3.0	3653	46.2%	78	11.3%
	3.0–5.9	1809	22.9%	75	10.9%
	6.0–8.9	1160	14.7%	89	12.9%
	9.0–11.9	772	0.1%	206	29.8%
	≥12	511	0.1%	243	35.8%
Weight	0–3.9 kg	503	6.4%	16	2.3%
	4–7.9 kg	1105	13.9%	73	10.6%
	8–11.9 kg	2354	29.8%	234	33.9%
	≥12	751	9.5%	87	12.6%
	N/A	3196	40.4%	281	40.7%

**Table 2:** The 10 most common disorders at the finest diagnostic precision recorded for Pugs that were euthanised and for Pugs that died non-assisted under primary-care veterinary practices participating in the VetCompass Programme in Australia from 1 January 2008 to 31 December 2017 (*n*  =  470).

Euthanised			Non-Assisted Deaths		
Fine-Level Diagnostic Term	Count	Percentage (%)	Fine-Level Diagnostic Term	Count	Percentage (%)
BOAS	29	8.1%	BOAS	8	7.1%
Seizure disorder	25	7.0%	Seizure disorder	8	7.1%
Degenerative spinal cord disorder	23	6.4%	Heat stroke	7	6.3%
Cognitive dysfunction	20	5.6%	Respiratory failure	5	4.5%
Neurological disorder (unspecified)	20	5.6%	Gastroenteritis	4	3.57%
Ulcerative keratitis	10	2.8%	Tick paralysis	4	3.6%
Osteoarthritis	8	2.2%	Cerebrovascular accident	3	2.7%
Intervertebral disc disorder	7	2.0%	Dog fight injury	3	2.7%
Mast cell tumour	7	2.0%	Drowned	3	2.7%
Diabetes mellitus	6	1.7%	Heart failure	3	2.7%
Grand Total	358		Grand Total	112	

**Table 3:** The top-level groupings recorded for Pugs that were euthanised and for Pugs that died non-assisted under primary-care veterinary practices participating in the VetCompass Programme in Australia from 1 January 2008 to 31 December 2017 (*n*  =  470).

Euthanised			Non-Assisted Deaths		
Top-Level Grouping	Count	Percentage	Top-Level Grouping	Count	Percentage
Neurological	106	29.6%	Respiratory	28	25.0%
Mass lesion	57	15.9%	Traumatic injury	15	13.4%
Respiratory	53	14.8%	Neurological	14	12.5%
Musculoskeletal	25	7.0%	Alimentary tract	13	11.6%
Ophthalmological	21	5.9%	Mass lesion	9	8.0%
Alimentary tract	19	5.3%	Cardiovascular	8	7.1%
Urogenital	14	3.9%	Intoxication	8	7.1%
Cardiovascular	12	3.4%	Urogenital	7	6.3%
Endocrine/metabolic	9	2.5%	Other condition	3	2.7%
Integumentary	9	2.5%	Haematopoietic	2	1.8%
Other condition	9	2.5%	Integumentary	2	1.8%
Traumatic injury	8	2.2%	Nutritional	2	1.8%
Intoxication	7	2.0%	Musculoskeletal	1	1.0%
Nutritional	4	1.1%			
Behavioural	3	0.8%			
Haematopoietic	2	0.6%			
Grand Total	358		Grand Total	112	

**Table 4:** Multivariate analysis of top disorder groups to demographic variables, identifying interaction terms. When one or more variable showed significance in the univariate regression model, they were included in the multivariate regression model. * Age and weight variables are per increment of 1 year and 1 kg, respectively.

Top-Level Grouping	Variable	Univariate Model (*p*-Value)	Multivariate Model (*p*-Value)
Mass Lesion	Neuter	0.001	0.081
	Age *	0.009	0.073
	Weight *	0.008	0.026
	Age:Weight *		0.023
Alimentary Tract	Age *	0.041	0.378
	Weight *	0.028	0.043
Traumatic Injury	Colour	6.422 × 10^−8^	0.000
	Neuter	1.194 × 10^−5^	0.013
	Age *	4.198 × 10^−7^	2.286 × 10^−5^
	Neuter:Age *		0.007

## Data Availability

Datasets are not publicly available records and were provided by VetCompass Australia to approved project participants, and all requests should be directed to VetCompass.

## Appendix A

### Appendix A.1

**Table A1:** Univariate analysis of major disorder groups to demographic variables including odds Ratio (OR) and 95% confidence interval.

Top-Level Grouping	Variable	*p*-Value	Odds Ratio	CI
Neurological (25.05%)	Colour	0.260	Fawn (base)	
	Black		0.828	0.67, 1.02
	Brown		0.788	0.634, 0.978
	Fawn		0.839	0.685, 1.027
	Silver		0.700	0.324, 1.512
	Sex	0.473	Female (base)	
	Male		0.979	0.925, 1.037
	Neuter	0.655	Entire (base)	
	Desexed		0.986	0.926, 1.05
	Age	**0.049**		
	Per increment of 1 year		1.007	1, 1.013
	Weight	0.739		
	Per increment of 1 kg		1.002	0.991, 1.014
Respiratory (17.52%)	Colour	0.274		
	Black		0.860	0.714, 1.035
	Brown		0.892	0.737, 1.079
	Fawn		0.844	0.706, 1.008
	Silver		0.751	0.381, 1.483
	Sex	0.751		
	Male		0.992	0.944, 1.042
	Neuter	0.638		
	Desexed		0.987	0.935, 1.042
	Age	0.349		
	Per increment of 1 year		0.997	0.992, 1.003
	Weight	0.729		
	Per increment of 1 kg		0.998	0.99, 1.007
Mass Lesion (14.26%)	Colour	0.976		
	Black		1.047	0.883, 1.243
	Brown		1.034	0.867, 1.234
	Fawn		1.033	0.877, 1.218
	Silver		0.931	0.498, 1.742
	Sex	0.101		
	Male		1.038	0.993, 1.086
	Neuter	**0.002**		
	Desexed		1.083	1.032, 1.138
	Age	**0.009**		
	Per increment of 1 year		1.007	1.002, 1.012
	Weight	**0.008**		
	Per increment of 1 kg		1.011	1.003, 1.019
Alimentary Tract	Colour	0.908		
(6.72%)	Black		1.052	0.933, 1.186
	Brown		1.040	0.919, 1.177
	Fawn		1.054	0.939, 1.182
	Silver		1.000	0.644, 1.553
	Sex	0.792		
	Male		1.004	0.973, 1.037
	Neuter	0.8		
	Desexed		1.005	0.97, 1.04
	Age	**0.041**		
	Per increment of 1 age		0.996	0.993, 1
	Weight	**0.028**		
	Per increment of 1 kg		0.993	0.987, 0.999
Musculoskeletal	Colour	0.72		
(5.70%)	Black		1.034	0.928, 1.153
	Brown		1.068	0.955, 1.194
	Fawn		1.039	0.936, 1.154
	Silver		1	0.672, 1.489
	Sex	**0.044**		
	Male		1.031	1.001, 1.061
	Neuter	0.616		
	Desexed		1.008	0.976, 1.041
	Age	0.132		
	Per increment of 1 year		1.003	0.999, 1.006
	Weight	0.739		
	Per increment of 1 kg		1.001	0.995, 1.007
Traumatic Injury	Colour	**0.000**		
(4.68%)	Black		1.043	0.950
	Brown		1.068	0.97, 1.175
	Fawn		1.021	0.933, 1.116
	Silver		2.718	1.932, 3.824
	Sex	0.7776		
	Male		1.004	0.977, 1.031
	Neuter	**0.000**		
	Desexed		0.937	0.91, 0.965
	Age	**0.000**		
	Per increment of 1 year		0.992	0.989, 0.995
	Weight	0.5725		
	Per increment of 1 kg		0.999	0.994, 1.003

Bold indicates *p* < 0.05.

## References

[1] American Kennel Club A. Pug. https://www.akc.org/dog-breeds/pug/.

[2] Teng K.T., McGreevy P.D., Toribio J.-A.L., Dhand N.K. (2016). Trends in popularity of some morphological traits of purebred dogs in Australia. Canine Genet. Epidemiol..

[3] O’Neill D.G., Darwent E.C., Church D.B., Brodbelt D.C. (2016). Demography and health of Pugs under primary veterinary care in England. Canine Genet. Epidemiol..

[4] Archer J., Monton S. (2011). Preferences for infant facial features in pet dogs and cats. Ethology.

[5] Australian National Kennel Club Pug. http://ankc.org.au/Breed/Detail/31.

[6] Australian Veterinary Association, Brachycephalic Dog Breeding. https://www.ava.com.au/policy-advocacy/policies/companion-animals-health/brachycephalic-dog-breeding/.

[7] O’Neill D.G., Sahota J., Brodbelt D.C., Church D.B., Packer R.M., Pegram C. (2022). Health of Pug dogs in the UK: Disorder predispositions and protections. Canine Med. Genet..

[8] Ladlow J., Liu N.-C., Kalmar L., Sargan D. (2018). Brachycephalic obstructive airway syndrome. Vet. Rec..

[9] O’Neill D.G., Jackson C., Guy J.H., Church D.B., McGreevy P.D., Thomson P.C., Brodbelt D.C. (2015). Epidemiological associations between brachycephaly and upper respiratory tract disorders in dogs attending veterinary practices in England. Canine Genet. Epidemiol..

[10] Mao J., Xia Z., Chen J., Yu J. (2013). Prevalence and risk factors for canine obesity surveyed in veterinary practices in Beijing, China. Prev. Vet. Med..

[11] Packer R.M., Shihab N.K., Torres B.B., Volk H.A. (2016). Risk factors for cluster seizures in canine idiopathic epilepsy. Res. Vet. Sci..

[12] Such Z.R., German A.J. (2015). Best in show but not best shape: A photographic assessment of show dog body condition. Vet. Rec..

[13] Packer R.M.A., O’Neill D.G., Fletcher F., Farnworth M.J. (2019). Great expectations, inconvenient truths, and the paradoxes of the dog-owner relationship for owners of brachycephalic dogs. PLoS ONE.

[14] Adams V.J., Evans K.M., Sampson J., Wood J.L. (2010). Methods and mortality results of a health survey of purebred dogs in the UK. J. Small Anim. Pract..

[15] Bonnett B.N., Egenvall A., Hedhammar A., Olson P. (2005). Mortality in over 350,000 insured Swedish dogs from 1995-2000: I. Breed-, gender-, age- and cause-specific rates. Acta Vet. Scand..

[16] O’Neill D.G., Church D.B., McGreevy P.D., Thomson P.C., Brodbelt D.C. (2013). Longevity and mortality of owned dogs in England. Vet. J..

[17] Proschowsky H.F., Rugbjerg H., Ersbøll A.K. (2003). Mortality of purebred and mixed-breed dogs in Denmark. Prev. Vet. Med..

[18] Fleming J., Creevy K., Promislow D. (2011). Mortality in North American dogs from 1984 to 2004: An investigation into age-, size-, and breed-related causes of death. J. Vet. Intern. Med..

[19] Erlen A., Potschka H., Volk H.A., Sauter-Louis C., O’Neill D.G. (2018). Seizure occurrence in dogs under primary veterinary care in the UK: Prevalence and risk factors. J. Vet. Intern. Med..

[20] Skovola E.V., Cherubini G.B.E. (2023). Neurological Disorders in Pug Dogs: A Retrospective Study of 285 Cases (2013–2020) from a Single Referral Hospital in the United Kingdom. J. Vet. Sci. Res..

[21] van Renen J., Kehl A., Buhmann G., Matiasek L.A., Zablotski Y., Fischer A. (2024). Allele frequency of a genetic risk variant for necrotizing meningoencephalitis in pug dogs from Europe and association with the clinical phenotype. Front. Vet. Sci..

[22] Nicholas F.W., Tammen I., Sydney Informatics Hub (2024). OMIA:001470-9615: Online Mendelian Inheritance in Animals (OMIA) [dataset]. https://omia.org/.

[23] Bertram S., ter Haar G., De Decker S. (2018). Caudal articular process dysplasia of thoracic vertebrae in neurologically normal French bulldogs, English bulldogs, and Pugs: Prevalence and characteristics. Vet. Radiol. Ultrasound.

[24] Nishida H., Nakata K., Maeda S., Kamishina H. (2019). Prevalence and pattern of thoracolumbar caudal articular process anomalies and intervertebral disk herniations in pugs. J. Vet. Med. Sci..

[25] Ryan R., Gutierrez-Quintana R., Haar G.t., De Decker S. (2019). Relationship between breed, hemivertebra subtype, and kyphosis in apparently neurologically normal French bulldogs, English bulldogs, and pugs. Am. J. Vet. Res..

[26] Johansson E. (2019). Genetic Variation in Genes Associated with Brachycephaly. Master’s Thesis.

[27] Driver C.J., Rose J., Tauro A., Fernandes R., Rusbridge C. (2019). Magnetic resonance image findings in pug dogs with thoracolumbar myelopathy and concurrent caudal articular process dysplasia. BMC Vet. Res..

[28] Mochizuki H., Motsinger-Reif A., Bettini C., Moroff S., Breen M. (2017). Association of breed and histopathological grade in canine mast cell tumours. Vet. Comp. Oncol..

[29] McNiel E., Prink A., O’Brien T. (2006). Evaluation of risk and clinical outcome of mast cell tumours in pug dogs. Vet. Comp. Oncol..

[30] McGreevy P., Thomson P., Dhand N.K., Raubenheimer D., Masters S., Mansfield C.S., Baldwin T., Soares Magalhaes R.J., Rand J., Hill P. (2017). VetCompass Australia: A National Big Data Collection System for Veterinary Science. Animals.

[31] Fawcett A., Barrs V., Awad M., Child G., Brunel L., Mooney E., Martinez-Taboada F., McDonald B., McGreevy P. (2019). Consequences and management of canine brachycephaly in veterinary practice: Perspectives from Australian veterinarians and veterinary specialists. Animals.

[32] Tambalis K., Panagiotakos D., Arnaoutis G., Psarra G., Maraki M., Mourtakos S., Grigorakis D., Sidossis L.S. (2015). Establishing cross-sectional curves for height, weight, body mass index and waist circumference for 4-to 18-year-old Greek children, using the Lambda Mu and Sigma (LMS) statistical method. Hippokratia.

[33] Venom Coding Group (2024). Venom Codes. https://venomcoding.org/venom-codes/.

[34] Downing F., Gibson S. (2018). Anaesthesia of brachycephalic dogs. J. Small Anim. Pract..

[35] Bruchim Y., Klement E., Saragusty J., Finkeilstein E., Kass P., Aroh I. (2006). Heat stroke in dogs: A retrospective study of 54 cases (1999-2004) and analysis of risk factors for death. J. Vet. Int. Med..

[36] Riggs J., Liu N.C., Sutton D.R., Sargan D., Ladlow J.F. (2019). Validation of exercise testing and laryngeal auscultation for grading brachycephalic obstructive airway syndrome in pugs, French bulldogs, and English bulldogs by using whole-body barometric plethysmography. Vet. Surg..

[37] Liu N.C., Adams V., Kalmar L., Ladlow J., Sargan D. (2016). Whole-body barometric plethysmography characterizes upper airway obstruction in 3 brachycephalic breeds of dogs. J. Vet. Intern. Med..

[38] Aromaa M., Lilja-Maula L., Rajamäki M. (2019). Assessment of welfare and brachycephalic obstructive airway syndrome signs in young, breeding age French Bulldogs and Pugs, using owner questionnaire, physical examination and walk tests. Anim. Welf..

[39] Jones S.A., Kennedy S.C. (2024). Comparison of mortality of brachycephalic dogs undergoing partial staphylectomy using conventional incisional, carbon dioxide laser, or bipolar vessel sealing device. Vet. Surg..

[40] Inoue M., Hasegawa A., Hosoi Y., Sugiura K. (2015). A current life table and causes of death for insured dogs in Japan. Prev. Vet. Med..

[41] Bennett P.F., Taylor R., Williamson P. (2018). Demographic risk factors for lymphoma in Australian dogs: 6201 cases. J. Vet. Intern. Med..

[42] Langenbach A., McManus P.M., Hendrick M.J., Shofer F.S., Sorenmo K.U. (2001). Sensitivity and specificity of methods of assessing the regional lymph nodes for evidence of metastasis in dogs and cats with solid tumors. J. Am. Vet. Med. Assoc..

[43] Inoue M., Kwan N.C., Sugiura K. (2018). Estimating the life expectancy of companion dogs in Japan using pet cemetery data. J. Vet. Med. Sci..

[44] Urfer S.R. (2011). Bias in canine lifespan estimates through right censored data. J. Small Anim. Pract..

[45] Michell A.R. (1999). Longevity of British breeds of dog and its relationships with sex, size, cardiovascular variables and disease. Vet. Rec..

[46] Edney A. (1998). Reasons for the Euthanasia of Dogs and Cats.

[47] McMullen S., Clark W., Robertson I. (2001). Reasons for the euthanasia of dogs and cats in veterinary practices. Aust. Vet. Pract..

[48] Salt C., Morris P.J., German A.J., Wilson D., Lund E.M., Cole T.J., Butterwick R.F. (2017). Growth standard charts for monitoring bodyweight in dogs of different sizes. PLoS ONE.

